# Targeting the nuclear orphan receptor NR4A1: a key target in lung cancer progression and therapeutic resistance

**DOI:** 10.3389/fonc.2025.1566598

**Published:** 2025-07-01

**Authors:** Minhan Jin, Yuhui Wang, Mingze Song, Wenfei Guo, Shirong Li, Zeqing Pu

**Affiliations:** 1 Department of Clinical Laboratory, WeiFang People’s Hospital, Shandong Second Medical University, Weifang, China; 2 School of Medical Laboratory, Shandong Second Medical University, Weifang, China

**Keywords:** NR4A1, lung cancer, molecular mechanism, DIM-C-pPhOH, cytosporone B, signaling pathways

## Abstract

As a malignant tumor with high morbidity and mortality, lung cancer is associated with a variety of risk factors, including smoking, exposure to occupational carcinogens, familial inheritance, and chronic lung disease. Lung cancer is often detected late and has a complex pathogenesis, so early diagnosis and intervention of lung cancer are essential. Finding effective targets is important to develop new treatments for lung cancer. As a member of Group 4A of the nuclear receptor subfamily, Nuclear Receptor Subfamily 4 Group A Member 1 (NR4A1) is an immediate early gene that encodes a transcription factor that plays a regulatory role when the cell and tissue microenvironment changes. NR4A1 plays a pro-cancer role in solid tumors including lung cancer, but a tumor suppressor role in hematological malignancies. NR4A1 palys a role through multiple mechanisms in lung cancer, including promoting cell proliferation by forming a complex with p300/specific protein 1 (Sp1) and acting on the survivin and AMP-activated protein kinase (AMPK)/mechanistic Target of Rapamycin Complex 1 (mTORC1) pathways, promoting metastasis and invasion by inducing the occurrence of transforming growth factor-β (TGF-β) dependent epithelial-mesenchymal transition (EMT), promoting vascular remodeling by acting on vascular endothelial growth factor A (VEGF-A), promoting immune escape by acting on programmed cell death-1 (PD-1) dependent T cell exhaustion, promoting cell apoptosis interacted with B-cell lymphoma-2 (Bcl-2) and promoting metabolic reprogramming by increasing fatty acid oxidation. In recent years, several studies on NR4A1-related agonists and inhibitors in lung cancer have been reported. These compounds are expected to become drugs for targeted tumor therapy, but current research is limited to cellular and animal experiments. It still takes time to verify and evaluate clinical applications, other biological effects and potential side effects. This review summarizes the biological role of NR4A1 in lung cancer and describes the molecular mechanisms and signaling pathways regulated by NR4A1. This paper will provide a theoretical basis for the early treatment of lung cancer by using NR4A1-related compounds in the clinic.

## 1 Introduction

According to global cancer statistics, in 2022, the number of lung cancer cases reached 2.481 million cases, accounting for 12.4% of cancers worldwide. Lung cancer accounts for 18.7% of all cancer deaths, ranking first among all types of cancer (1). The American Cancer Society 2025 model estimates 226,650 new cases of lung cancer and 124,730 deaths in the United States in 2025 (2). Its high incidence and mortality are important issues in the field of global public health. From a histologic point of view, lung cancer is mainly classified into two major types: small cell lung carcinoma (SCLC) and non-small cell lung carcinoma (NSCLC). NSCLC accounts for about 80%–85% of lung cancer cases and can be further subdivided into three main subtypes: adenocarcinoma, squamous cell carcinoma, and large cell carcinoma (3). Lung cancer is highly insidious, so most of the patients are in the middle to late stage when they are diagnosed, and the primary tumor is often accompanied by localized or distant metastasis. The treatment strategies for lung cancer include surgical resection (4), chemotherapy (5), radiotherapy (6), targeted therapy (7, 8), immunotherapy (9, 10), nano drug delivery therapy system (11), molecular targeted treatment system (12), photothermal treatment strategy (13). Despite the various treatment modalities, the prognosis of lung cancer is still poor and the five-year survival rate of patients is relatively low. The search for new treatment methods for lung cancer and the identification of key target genes have become important tasks that need to be solved urgently.

Recent studies have found that nuclear receptor NR4A1 is widely expressed in a variety of tumors, including lung cancer. NR4A1, a transcription factor, regulates related signaling pathways and participates in tumor cell proliferation (14), migration (15), invasion (16), apoptosis (17) and immunoregulation (18). The aim of this article is to summarize the regulatory role and mechanism of NR4A1 in lung cancer to provide a sound theoretical basis for the clinical treatment of lung cancer.

## 2 Structure and role in oncology of NR4A1

**Figure 1:**
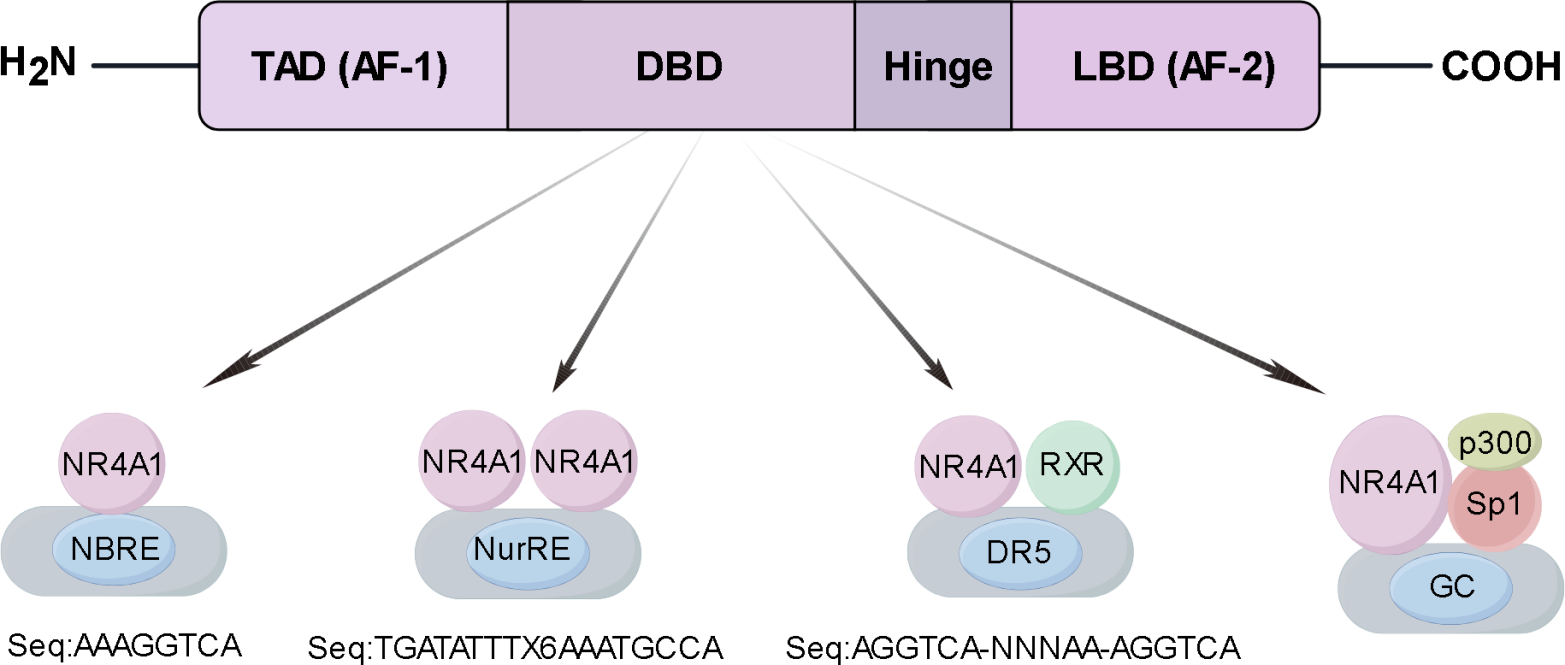
A NR4A1 structure and functional domains schematic. Structure of NR4A1 including DNA-binding domain (DBD), C-terminal ligand-binding domain (LBD), hinge region (Hinge), and N-terminal trans-activating domain (TAD). The DBD region interacts specifically with the DNA sequences of the NBRE and the NurRE. NR4A1 forms a heterodimer with the retinoid X receptor (RXR), which then binds to the DR5 response element to produce transcriptional activation. NR4A1 forms DNA-binding complexes with Sp1 and p300 to exert transcriptional.

NR4A1 (Nuclear Receptor subfamily 4 group A member 1, also known as Nur77/TR3) is an early stress gene that acts as an important transcription factor to regulate the expression of multiple target genes (19). NR4A1 is also thought to mediate tumor metabolism (20). In addition to NR4A1, the NR4A nuclear receptor subfamily also includes NR4A2 and NR4A3. The three exhibits similar structures, including a DNA-binding domain (DBD), C-terminal ligand binding domain (LBD) and N-terminal trans-activation domain (TAD) (**Figure 1**). The intermediate DBD can interact specifically with the DNA sequence of NBRE and NurRE. The TAD contains the ligand-independent activation function 1 (AF-1) region, which regulates the activity of transcription factors. The DBD can form a response element with NBRE (AAAGGTCA) or interact with NurRE (TGATATTTX6AAATGCCA) DNA sequences as a homodimer or heterodimer (21). NR4A1 forms a heterodimer with the retinoid X receptor (RXR), which then binds to the DR5 response element to produce transcriptional activation (sequence: AGGTCA-NNNAA-AGGTCA) (22, 23). In addition to the three binding modes mentioned above, NR4A1 forms DNA-binding complexes with Sp1 and p300 to exert transcriptional activation in lung and pancreatic cancer cells (24, 25). The LBD contains the ligand-dependent activation function 2 (AF-2) region, which recognizes the corresponding ligand to ensure transcriptional activity (26, 27).

**Figure 2:**
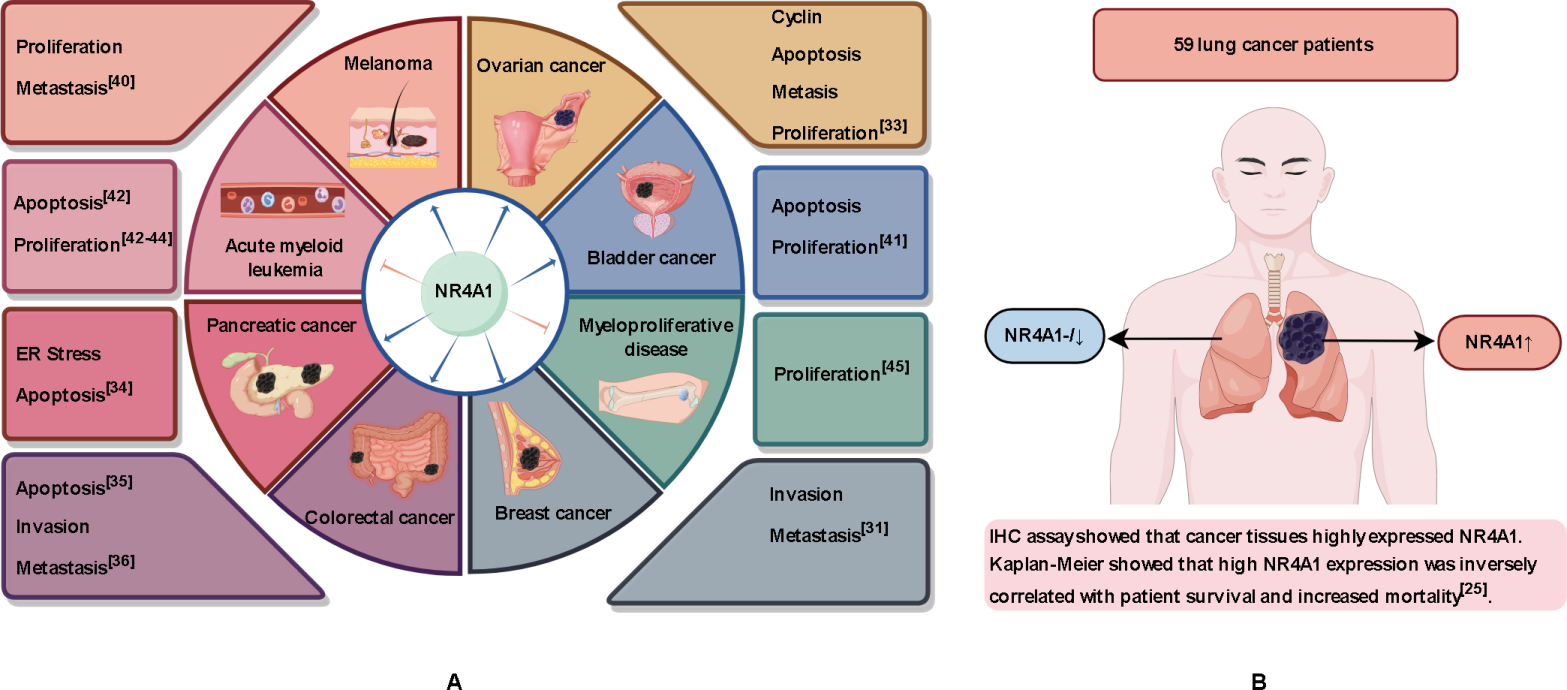
Role of NR4A1 in different tissues. **(A)** NR4A1 plays a tumor-promoting role in lung, breast, colorectal, pancreatic, melanoma, and bladder cancers. NR4A1 exerts an inhibitory effect in acute myeloid leukemia and myeloproliferative diseases. **(B)** NR4A1 expression is normal or low in tissues of healthy individuals and in tissue adjacent to the tumor in lung cancer patients, but NR4A1 is overexpressed in cancerous tissues.

NR4A1 was initially characterized as a gene inducible by serum growth factors, and its overexpression has been found in a variety of solid tumors (28–30). In breast cancer, inflammatory factors can induce NR4A1 expression both *in vivo* and *in vitro*. Whole-genome cDNA screening results showed that nuclear receptor NR4A1 is a strong activator of transforming growth factor-β (TGF-β) signaling, which can enhance the migration, invasion, and metastasis of breast cancer cells (31). The long non-coding RNA MALAT1 modulates NR4A1 expression through a downstream regulatory element in breast cancer cells (32). NR4A1 is highly expressed in high-grade serous ovarian cancer samples with poor progression-free survival and is mainly localized in the cytoplasm and nucleus (33). NR4A1 regulates endoplasmic reticulum stress and Reactive oxygen species (ROS) levels in pancreatic cancer cells to promote cell proliferation and survival (34). Immunohistochemical staining of 20 colon tumors and 20 normal colon tissues showed that the proportion of colon tumors with high NR4A1 staining was as high as 60% (12/20), while that of normal colon tissues was only 10% (2/20) (35). NR4A1 promotes invasion and metastasis of colorectal cancer cells by up-regulating matrix metalloproteinase-9 and subsequently down-regulating E-cadherin (36). Cheng et al. findings suggest that targeting NR4A1 with OSI-930 may be a promising therapeutic strategy for COAD patients with high levels of immune infiltration (37). Although NR4A1 expression promotes the growth of these tumors, NR4A1 regulated by long non-coding RNAs activates the apoptosis signaling pathway and inhibits the progression of endometrioid endometrial carcinoma (38). NR4A1 is also required for melanoma growth (39). Phosphoserine phosphatase reduces 2-hydroxyglutarate levels and inhibits histone demethylases in melanoma cells, upregulates NR4A1 expression and promotes tumor growth and metastasis (40). NR4A1 also plays an important role in the developmental process of bladder cancer (41). In addition, NR4A1 showed opposite effects in hematologic tumors compared with solid tumors. Low expression of NR4A1 has been found in acute myeloid leukemia (42–44) and chronic myelodysplastic/myeloproliferative diseases (45), where it exhibits tumor-suppressive effects (**Figure 2A**). These conflicting roles of NR4A1 that NR4A1 may play different roles depending on the type and location of the cancer.

## 3 Correlation between NR4A1 expression and lung cancer

The expression level of NR4A1 varies with the subcellular localization. NR4A1 plays a multi-effect regulatory role and although it is generally accepted that NR4A1 has a pro-cancer effect, the correlation between high expression and adverse clinical outcomes is controversial. Seong et al. collected RNA-seq data from 1013 lung cancer cases and 397 normal tissue samples from the TCGA and GTEx databases and found that NR4A1 expression was lower in lung adenocarcinoma (LUAD) and lung squamous cell carcinoma (LUSC) than in normal tissue (46). Huang et al. similarly performed RNA-seq using tumor and tumor-adjacent tissues from four LUAD patients and found low expression of NR4A1 in the cancer tissues (47). We searched the GEPIA and TissGDB databases and found that the expression of NR4A1 was significantly reduced in LUAD and LUSC. However, Lee et al. collected tissue from 59 patients with NSCLC and adjacent normal lung tissue and, by immunohistochemical analysis, showed that NR4A1 was expressed in lung cancer but there was low or no expression in normal lung tissue (**Figure 2B**). Overexpression of NR4A1 is associated with reduced survival and poor clinical outcomes in patients with NSCLC (25). Yang et al. used the STRING database to show that NR4A1 expression correlates with RNA polymerase I subunit B (POLR1B) activity, and POLR1B is an important modulator of lung cancer cell proliferation (48). Although the role of NR4A1 in lung cancer remains to be verified, a growing number of studies have found that NR4A1 plays a pro-cancer role in lung cancer (49–51).

## 4 Role and molecular mechanism of NR4A1 in the pathogenesis of lung cancer

**Figure 3:**
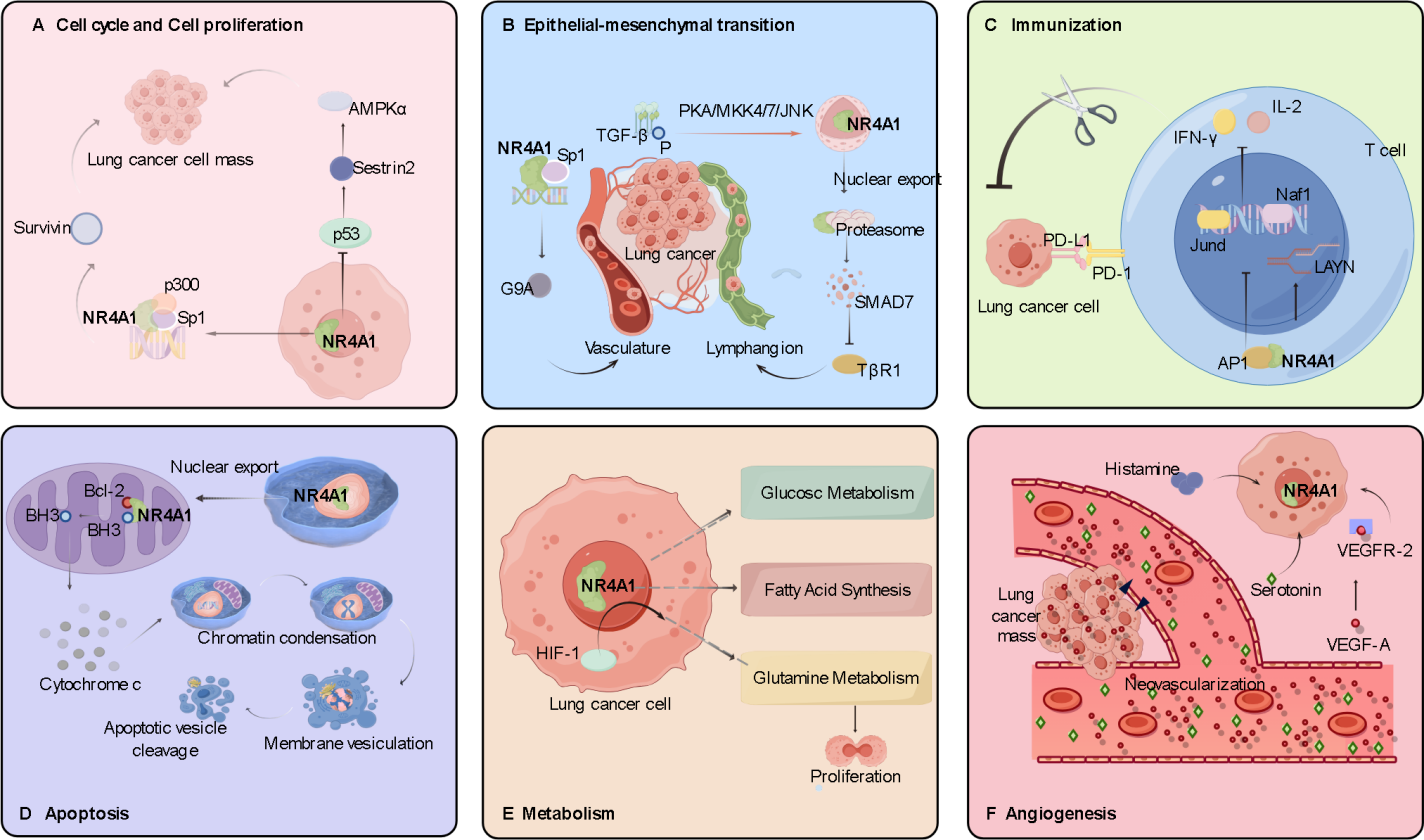
Role and molecular mechanism of NR4A1 in the pathogenesis of lung cancer. **(A)** The role of NR4A1 in lung cancer cell proliferation. The NR4A1/Sp1/p300 complex upregulates survivin; NR4A1 inhibits p53 acetylation, which in turn induces activation of the AMPK/mTORC1 pathway. **(B)** The role of NR4A1 in lung cancer cell migration and invasion. The NR4A1/Sp1 complex promotes G9A expression; NR4A1 export from the nucleus is induced by phosphorylation of the TGF-β/TGF-β receptor. **(C)** The role of NR4A1 in immunomodulation. The NR4A1/AP-1 complex decreases tumor cell killing by T cells by reducing IFN-γ and IL-2 secretion. **(D)** The role of NR4A1 in apoptosis of lung cancer cells. Nuclear export of NR4A1 indirectly induces apoptosis by NR4A1 binding to mitochondrial Bcl-2. **(E)** The role of NR4A1 in lung cancer cell metabolism. **(F)** The pro-angiogenic role of NR4A1 in lung cancer cells. VEGF-A binding to VEGF-A receptor 2 upregulates NR4A1 expression and participates in angiogenesis.

The role of NR4A1 in lung cancer involves multiple aspects, including transcriptional regulation, protein-protein interactions, and post-translational modifications. NR4A1 is regulated at both the transcriptional and post-transcriptional levels and regulates downstream signaling pathways involved in lung cancer processes, including angiogenesis, cell proliferation, migration, invasion, apoptosis, and immune regulation (**Figure 3**). The role of NR4A1 also depends on the subcellular localization, expression level, and co-activator/co-repressor factors. NR4A1 interferes with intracellular regulation at different levels through various signaling pathways associated with many cancers, and understanding these interactions may elucidate the role of this family member in tumorigenesis and tumor suppression.

### 4.1 Cell cycle and cell proliferation

Molecules that inhibit tumor cell proliferation or cell cycle checkpoints play an important role in alleviating tumor progression. Stimulation by serum and epidermal growth factor induces trans-activation of NR4A1 in H460 and Calu-6 lung cancer cells, and high expression of NR4A1 promotes cell cycle progression, exerting a positive effect on mitosis in lung cancer cells (49). Zhang et al. found that NR4A1 promotes cell mitosis and survival via its transcriptional activity in the nucleus (50). Lee’s group found that NR4A1 promotes the proliferation of lung cancer cells in two ways. The first involves the expression of p300, which has histone acetyltransferase activity. In A549 and H460 cells, p300 enhances NR4A1 acetylation and protein stability. NR4A1 interacts with specific protein 1 (Sp1) or specific protein 4 (Sp4) to form the NR4A1-Sp1/Sp4-p300 DNA-binding complex. This complex binds to GC-rich promoters and upregulates survivin expression, thereby promoting lung cancer cell survival. The second way that NR4A1 promotes lung cancer cell proliferation is by inhibiting p53 expression to induce Adenosine 5’-monophosphate (AMP)-activated protein kinase (AMPK)/mechanistic Target of Rapamycin Complex 1 (mTORC1) pathway activation (25).

### 4.2 Epithelial-mesenchymal transition

Tumor cell metastasis is the main cause of lung cancer patient mortality. Tumor cells can acquire invasive ability through epithelial-mesenchymal transition (EMT), after which they invade vascular or lymphatic vessels and ultimately distant organs. The high metastasis and mortality rates of lung cancer are related to EMT in lung tumors (51–53). TGF-β acts as a multifunctional regulator of migration and upregulates the expression of key EMT regulators, such as Snail and δEF1/SIP1 (51, 54, 55). Erik’s group found that TGF-β-induced migration depends on the nuclear export of NR4A1 in cell lines such as A549, H460, and H1299, and the migration could be blocked by the NR4A1 inhibitor DIM-C-pPhOH (56). Paraspeckle Component 1 (PSPC1) is an activator of TGF-β-dependent EMT. Safe et al. found that NR4A1, by acting as a transcription factor, enhances the promoter sequence activation of the PSPC1 gene, thereby promoting EMT (57). Fan et al. similarly concluded that the nuclear long non-coding RNA (lncRNA) LETS1 inhibits SMAD7-induced TGF-β type I receptor polyubiquitylation through activation of NR4A1 expression in A549 cells, thereby promoting TGF-β-induced EMT migration and extravasation of cancer cells (58).

### 4.3 Angiogenesis

Tumor growth requires the formation of new blood vessels to deliver oxygen and nutrients. Vascular endothelial growth factor (VEGF) is a highly specific pro-vascular endothelial cell growth factor, which plays a key role in tumorigenesis and development (59). Using a DNA microarray assay, Zeng et al. found that NR4A1 gene expression was upregulated in human umbilical vein endothelial cells (HUVECs), and knockdown of NR4A1 limited the effect of VEGF-A on HUVECs and inhibited tumor angiogenesis (60). It has also been reported that histamine and serotonin play positive roles in angiogenesis Previously, it was also reported that histamine and serotonin play positive roles in angiogenesis (61). Qin et al. implanted histamine or serotonin pellets subcutaneously in wild-type mice and found that both induced angiogenesis in a dose-dependent manner, but little angiogenesis occurred in NR4A1^-/-^ mice (62).

### 4.4 Immunomodulation

Immunomodulation is involved in the entire process of tumorigenesis and development. Tumor cells can evade immune surveillance as well as resist immune defense in various ways, such as through gene mutation or tumor antigen defects. In recent years, more and more studies have shown that NR4A1 can help tumor cells achieve immune escape by affecting the function of immune cells in the tumor microenvironment (TME), and then regulate tumor development (63–66).

In the immune system, NR4A1 regulates the immune response mainly by inhibiting the recognition and proliferation of T cells, thus reducing the body’s ability to monitor and attack lung cancer cell. Liu et al. identified NR4A1 as a key molecule in T cell dysfunction by genome-wide analysis, and NR4A1 was stably highly expressed in tolerant T cells (Ttol). In T cells, overexpression of NR4A1 in combination with activating protein-1 (AP-1) inhibits the expression of effector genes, such as *Jund* and *Naf1*, which reduces their secretion of interferon-gamma (IFN-γ) and Interleukin-2 (IL-2), leading to a significant decrease in the tumor-killing effect of T cells (67). Yang et al. also found that NR4A1 upregulates the *LAYN* gene at the transcriptional level, thereby inhibiting the killing function of CD8^+^ T cells against LUAC (68). Sana et al. found that tumor growth was significantly inhibited in Mice lacking NR4A1 and NR4A2 genes specifically in Tregs subcutaneously inoculated with Lewis lung carcinoma cells, and inhibition of NR4A1 in tumor-infiltrating regulatory T cells (TI-Tregs) breaks down the immune tolerance to tumor cells and promotes the antitumor activity of tumor-infiltrating CD8^+^ T cells (69).

In recent years, chimeric antigen receptor (CAR) T cell therapy has been widely used in leukemia and lymphoma but is less effective in solid tumors, such as lung cancer. NR4A1 promotes the expression of inhibitory receptors, such as programmed cell death-1 (PD-1), leading to the depletion or dysfunction of CAR T cells, which ultimately allows lung cancer cells to evade the immune response (70, 71). Kensuke et al. transferred NR4A1/2/3 gene knockout CAR T-cells into A549 tumor-bearing immunodeficient mice and reached a similar conclusion (72). The above studies indicate that NR4A1 depletes T cells, inhibiting the proliferation and killing function of T cells, and is thus a promising tumor immunotherapy target for mediating T cells.

### 4.5 Apoptosis

NR4A1 plays a pro-apoptotic role in a variety of cancers, in part because of its localization in the nucleus. When NR4A1 translocates from the nucleus to the mitochondria, it interacts directly with the Bcl-2 protein and change the Bcl-2 conformation, exposing the pro-apoptotic BH3 domain, which triggers the release of cytochrome c and indirectly induces apoptosis (73–81). TIAM1, a small GTPase RAC1 activator, interacts with NR4A1 in the nucleus of SCLC cells to reduce their cell viability and tumorigenicity. Malayoside, an extract from *Antiaris toxicaria* Lesch, activates ERK1/2 and p38 and phosphorylates NR4A1 in H460 cells, prompting NR4A1 to translocate to the mitochondria (82). NR4A1 also binds to the promoter of the anti-apoptotic protein BRE, exerting a pro-apoptotic effect by interfering with BRE function (83). Martin et al. found that NR4A1-derived peptides can induce apoptosis in paclitaxel-resistant cancer cells by acting on Bcl-2 (84). Liu et al. Found that quinoline derivative 10E modulates the pro-apoptotic nuclear export of NR4A1 in A549 and H460 cells (85). These findings suggest that the effect of NR4A1 on Bcl-2 provides a theoretical basis for targeted cancer therapy.

### 4.6 Metabolic reprogramming

Tumor cells provide substrates and energy for themselves through metabolic reprogramming activities, such as glycolysis, glutamine metabolism, fatty acid metabolism, and nucleic acid and amino acid metabolism, to promote tumor cell activity. NR4A family receptors are considered mediators of metabolic markers in tumors. Holla et al. found that NR4A1 plays a pro-oncogenic role in the regulation of fatty acid oxidation pathways in colon cancer (86). In breast cancer and melanoma, Poirot et al. found that the cholesterol metabolite dendrogenin A activates NR4A1 expression and exhibits tumor suppressor effects (87). Dysregulation of glutamine metabolism occurs in a variety of solid tumor cells and is essential for cancer cell proliferation (88). Hypoxia-inducible factor 1 (HIF-1) is an important regulator of glutamine metabolism, and Christoph et al. found that the expression of HIF-1 and NR4A1 was upregulated in A549 cells (89). The expression of NR4A1 in metabolic pathways is expected to inhibit the abnormal metabolism of tumor cells. NR4A1 potentially leads to targets for the control of tumor development.

## 5 Research progress of NR4A1 inhibitors or activators

Targeted therapies can attack tumor cells more precisely than chemotherapy and can reduce the incidental killing of normal cells. In recent years, studies have shown that targeted inhibition of NR4A1 can play an important role in preventing the development of lung cancer. In future clinical applications, in addition to the use of cisplatin, Nimotuzumab, and Bevacizumab alone in the treatment of lung cancer, the combination of NR4A1-targeted drugs with monoclonal antibody chemotherapy may provide a better treatment option for patients.

Cytosporone B (Csn-B), a natural agonist of NR4A1, specifically binds to the LBD region of NR4A1 and enhances the trans-activation of NR4A1. Wu’s laboratory found that Csn-B promotes the translocation of NR4A1 from the nucleus to the mitochondria, mediating the onset of apoptosis. They also measured the proliferative effects of Csn-B on several tumor cell lines and found that Csn-B inhibited the proliferation of BGC-823 human gastric cancer cells and SW620 human colon cancer cells by >70%, but inhibited H1299 human lung cancer cells and HepG2 human liver cancer cells by ≥40% (90). Dawson et al. showed that the interaction of Csn-B with the NR4A1 LBD region inhibited the viability of H460 lung cancer cells with a weaker efficiency than the positive control, DIM-Ph-4-CF3 (91). Amoitone B, a Csn-B analog nanocrystal, preferentially targets lung tissue and may be a potentially effective antitumor agent, although it has only a moderate inhibitory effect on H460 cells (92). The compound CCE9, which is extracted from Chinese herbal plants, can induce NR4A1 expression and Bcl-2 phosphorylation, leading to NR4A1 cytoplasmic localization and induction of the NR4A1-Bcl-2 apoptosis pathway in a p38α MAPK-dependent manner (93). The above results suggest that Csn-B and its derivatives have pro-apoptotic effects in a variety of tumor cells, but their antitumor effects are selective. Therefore, the inhibitory effect of Csn-B and its derivatives on lung cancer cells needs to be further studied.

DIM-C-pPhOH (C-DIM-8), a cruciferous plant-derived indole compound, and its derivatives are commonly employed in research exploring cancer cell proliferation and apoptotic pathways (94, 95). Lee et al. found that in lung cancer cells (A549, H460, and H1299), DIM-C-pPhOH reduced NR4A1 trans-activation and inactivated the NR4A1/p300/Sp1 complex, which in turn exhibited antitumor activity and low toxicity (25). Two C-DIM analogs, DIM-C-pPhOCH3 (C-DIM-5) and C-DIM-8, induced apoptosis in A549 cells, leading to a G0/G1-to-S phase block and tumor growth inhibition (96). Kumaravel found that C-DIM reduced PSPC1-mediated TGFβ cancer-promoting activity by inhibiting NR4A1, the upstream regulator of PSPC1 (57) Summary of the main targeted drugs is shown in **Table 1**.

**Table 1:** Research progress of NR4A1 inhibitors and agonists in lung cancer.

Category	Drug	Molecule/ pathway	Expression	Regulation effect	Method	Study subjects	Reference
Agonist	Cytosporone B	NR4A1(LBD)	Promotion	Inhibition of cell proliferation	MTT	BGC-823 C57BL/6	(90)
				Apoptosis promotion	Immunohistochemistry TUNEL assays		
		NR4A1(LBD)	/	Inhibition of cell proliferation	MTT	H460	(91)
				Apoptosis promotion	Immunohistochemistry		
	Amoitone B	G1 cycle arrest	/	Inhibition of cell proliferation	MTT	H460 BGC-823 HepG2 SW620/ Kunming strain mice	(92)
				Apoptosis promotion	Hoechst staining Flow cytometric analysis Annexin V-FITC/PI staining		
	CCE9	p38α MAPK NR4A1-Bcl-2	Promotion	Apoptosis promotion	Immunofluorescence Apoptosis assay Flow cytometric analysis	A549 HepG2 HeLa229	(93)
Suppressant	DIM-C-pPhOH	NR4A1/p300/ Sp1 p53/sestrin2/AMPKα	No Effect	Inhibition of cell proliferation	Cell proliferation assay *In vivo* experiments	A549 H460 H1299/ A549 -C57BL/6	(25)

In addition to C-DIM, coumarin derivative apaensin has been found to be an inducer targeting NR4A1, which has anticancer effects by regulating the NR4A1-Bcl-2 apoptotic pathway (97). A coumarin derivative extracted from Arrowwood Antiaris toxicaria (98), resveratrol extracted from fruits and vegetables (99), cardiac glycosides (100), malayoside (82), isoharringtonine (IHT) (101) and other compounds have similar effects on lung cancer cell and exert anticancer effects by inhibiting lung cancer cell growth, inducing NR4A1 nuclear export, and activating NR4A1-Bcl-2 apoptosis pathway. Other compounds are shown in **Table 2**.

**Table 2:** Research progress of other compounds of NR4A1 in lung cancer.

Category	Drug	Molecule/ Pathway	Expression	Regulation effect	Method	Study subjects	Reference
Other Compounds	Antiaris toxicaria	/	Promotion	Inhibition of cell proliferation	MTT	H460	(94)
				Apoptosis promotion	Apoptosis assay		
	Malayoside	ERK1/2 p38	Promotion	Inhibition of cell proliferation	MTT	H460 H252 A549	(81)
				Apoptosis promotion	Apoptosis assay		
	Isoharringtonine	/	No Effect	Inhibition of cell proliferation	Cell viability assay	A549 H460/ A549- NR4A1-/-C57BL/6	(98)
				Apoptosis promotion	Apoptosis assay		
	Cardiac glycosides	/	Promotion	Inhibition of cell proliferation	MTT	H460	(99)
	Apaensin	JNK P38 MAPK NR4A1-Bcl-2	Promotion	Apoptosis promotion	Immunofluorescence	H460 MCF-7	(100)

The above natural and synthetic compounds can affect the expression and action of NR4A1 and have a good prospect of becoming the basis of tumor-targeted therapy. However, the effect is limited to molecular, cellular, and animal experiments, and it will take time to verify and evaluate the clinical applications, other biological effects, and potential side effects.

## 6 Summary and prospect

According to our current understanding, NR4A1 plays an important role in the occurrence and development of lung cancer. NR4A1 is a potential antitumor drug target, and precise targeting of NR4A1 can induce tumor cell apoptosis and inhibit cell growth. However, there is still a long way to go in researching NR4A1 as a target for chemoprevention of lung cancer. By using single-cell RNA sequencing technology combined with spatial transcriptomics, the dynamic changes of heterogeneity within tumors can be analyzed, especially in the constantly evolving tumor microenvironment. Future research directions can be more inclined to search for molecular targets that address intratumoral heterogeneity. In addition, the role of NR4A1 in cancer depends on the degree of expression and the subcellular site and is characterized by tissue selectivity. The development of subcellular specific NR4A1 modulators may be a direction for future precision therapy.
