# Correction: Targeting the nuclear orphan receptor NR4A1: a key target in lung cancer progression and therapeutic resistance

**DOI:** 10.3389/fonc.2026.1821472

**Published:** 2026-03-18

**Authors:** Minhan Jin, Yuhui Wang, Mingze Song, Wenfei Guo, Shirong Li, Zeqing Pu

**Affiliations:** 1 Department of Clinical Laboratory, WeiFang People’s Hospital, Shandong Second Medical University, Weifang, China; 2 School of Medical Laboratory, Shandong Second Medical University, Weifang, China

**Keywords:** NR4A1, lung cancer, molecular mechanism, DIM-C-pPhOH, cytosporone B, signaling pathways

An incorrect number was provided for ZR202111160201. The correct number is ZR2022QH279.

The original version of this article has been updated.

